# *In vitro* exposure to clofazimine can select for delamanid and pretomanid resistance in *Mycobacterium tuberculosis*

**DOI:** 10.1128/aac.01113-25

**Published:** 2025-09-22

**Authors:** Praharshinie Rupasinghe, Nabila Ismail, Wim Mulders, Robin M. Warren, Lavania Joseph, Dumisani Ngcamu, Thabisile Gwala, Shaheed V. Omar, Jens Vereecken, Bouke C. de Jong, Leen Rigouts, Emanuele Borroni, Daniela M. Cirillo, Thomas Schön, Paolo Miotto, Claudio U. Köser

**Affiliations:** 1 Unit of Mycobacteriology, Department of Biomedical Sciences, Institute of Tropical Medicine 829633 , Antwerp, Belgium; 2 Belgian Coordinated Collections of Microorganisms – Mycobacterial collection (BCCM-ITM), Antwerp, Belgium; 3 South African Medical Research Council Centre for Tuberculosis Research, Division of Molecular Biology and Human Genetics, Faculty of Medicine and Health Sciences, Stellenbosch University 121470, Cape Town, South Africa; 4 Centre for Tuberculosis, National Institute for Communicable Diseases, a division of the National Health Laboratory Services 70687, Johannesburg, South Africa; 5 Emerging Bacterial Pathogens Unit, IRCCS San Raffaele Scientific Institute, Milan, Italy; 6 Department of Biomedical and Clinical Sciences, Division of Infection and Inflammation, Linkoping University 568027, Linköping, Sweden; 7 Department of Infectious Diseases, Kalmar County Hospital, Linköping University, Kalmar, Sweden; 8 Department of Infectious Diseases, Linköping University Hospital 56750, Linköping, Sweden; 9 Department of Genetics, University of Cambridge 98529, Cambridge, United Kingdom; City St George's, University of London, London, United Kingdom

**Keywords:** *Mycobacterium tuberculosis*, clofazimine, delamanid, pretomanid, antimicrobial resistance

## Abstract

*In vitro* experiments with *Mycobacterium tuberculosis* showed that clofazimine exposure selected for delamanid and pretomanid resistance and mutations in *fbiA*, *fbiC*, or *fbiD*—after the acquisition of *Rv0678* mutations where this could be determined. Whether this is also possible *in vivo* and in an *Rv0678* wild-type background has to be studied further. Based on the available evidence, however, we propose that nitroimidazole resistance should not be considered an exclusion criterion for the use of clofazimine.

## INTRODUCTION

Clofazimine is a group B drug for treating rifampicin-resistant *Mycobacterium tuberculosis* and is included in newly WHO-recommended 6- or 9-month all oral regimens (1). Mutations in *Rv0678*, a negative regulator of the MmpS5-MmpL5 efflux pump, represent the dominant clofazimine resistance mechanism known to date, whereas *pepQ* (*Rv2535c*) mutations are much rarer (both mechanisms confer cross-resistance to bedaquiline) (2). Although *Rv1979c* has been associated with clofazimine resistance in the literature, no mutations in this gene have been recognized as markers of clofazimine resistance by the WHO mutation catalog to date (2). Six non-essential genes (i.e., *ddn* [*Rv3547*], *fbiA* [*Rv3261*], *fbiB* [*Rv3262*], *fbiC* [*Rv1173*], *fbiD* [*Rv2983*], and *fgd1* [*Rv0407*]) are required for the activation of both delamanid and pretomanid and, consequently, usually confer cross-resistance to both nitromidazoles when inactivated (2, 3). Waller et al. were the first and, so far, only group to demonstrate that clofazimine can select for high-level *in vitro* pretomanid resistance in *M. tuberculosis* due to mutations in *fbiA* and *fbiC* (4). We set out to investigate this property of clofazimine further.

Waller et al. used an avirulent auxotrophic strain derived from the lineage 4 H37Rv as the parental strain and obtained mutations in *fbiA* and *fbiC* after a single round of selection using three different concentrations of clofazimine, whereas *Rv0678* remained wild-type (4). Based on CRISPR interference using the same *Rv0678* wild-type parental auxotroph, they then found that transcriptional repression of *fbiA*, *fbiB*, *fbiC*, *fbiD*, and *fgd1* confers approximately twofold MIC increases to clofazimine, whereas the pretomanid MIC increased between 5- to 40-fold depending on the gene (4). By contrast, the clofazimine MIC did not change when *ddn* expression was repressed, even though this repression correlated with a 46-fold MIC increase to pretomanid. These findings were mirrored in the bactericidal activity assays (4). The shared target of delamanid and pretomanid, *dprE2* (*Rv3791*), and other more recently described potential resistance genes were not investigated (5, 6). Although not formally tested by Waller et al., it is likely that these *fbiA* and *fbiC* mutants were cross-resistant to delamanid (2, 3).

In Ismail et al., we reported the findings of *in vitro* selection experiments involving passages of increasing clofazimine concentrations (7). When analyzing the population derived from the lineage 4 H37Rv ATCC 27294 reference strain after the fourth passage, we found that a non-synonymous mutation in *fbiC* and an *fbiD* deletion had likely been selected after the acquisition of an *Rv0678* frameshift (Table 1) (7). The equivalent experiment using the R1, a lineage 2 strain, yielded two different *Rv0678* mutations as well as two *fbiA* mutations, for which the order of acquisition could not be inferred (Table 1). Because our focus in Ismail et al. had been on bedaquiline and clofazimine resistance, no antimicrobial susceptibility testing for nitroimidazoles was done at the time (7). Since then, we determined the delamanid and pretomanid MICs to confirm that these *fbiA* and *fbiD* mutations correlate with cross-resistance to both agents using the latest WHO critical concentrations (Table 1).

**TABLE 1:** Antimicrobial susceptibility testing results of *in vitro* selected mutants following serial exposure to clofazimine by Ismail et al. (7)^*h*^

Parental strain	Acquired mutations and allele frequency^*a*^	MGIT MIC (µg/mL) and corresponding categorical AST result^*b*^					
		CFZ		DLM		PMD	
		Baseline	4th passage	Baseline	4th passage	Baseline	4th passage
H37Rv^*c*^	*Rv0678* 214dupC^*d*^ (1.0), *fbiC* Glu142Asp (0.1),^*e*^ *fbiD* 559_561delTGC (0.5)	0.125 S	4 R	0.125 S	>1 R	0.125 S	>2 R
R1^*f*^	*Rv0678* Gln22Pro (0.4) and 172_183delACGGCGCTGGCG (0.6), *fbiA* 285_286delTG^*g*^ (0.4) and Trp288Arg (0.3)	0.5 S	4 R	0.125 S	>1 R	0.25 S	>2 R

^*a*^ The figures in parentheses correspond to the allele frequency after four passages on clofazimine-containing media. Although no sequencing was carried out after any of the first three passages, the *fbiC* mutation and *fbiD* deletion in the H37Rv-derivative likely evolved after the *Rv0678* insertion because only the latter is fixed in the population. The order of evolution and genetic linkage of all *fbiA* and *Rv0678* mutations in the R1-derivative cannot be inferred.

^*b*^ Interpretation according to the current WHO critical concentrations (i.e., 0.06 µg/mL for delamanid and 0.5 and 2.0 µg/mL for pretomanid) were tested (8).

^*c*^ ATCC 27294. Lineage 4. Genome accession: ERS23863176.

^*d*^ Marker for bedaquiline and clofazimine cross-resistance according to the second edition of the WHO mutation catalog (2).

^*e*^ Not reported in Ismail et al. because an allele frequency of 0.3 was used as the cutoff (7).

^*f*^ Lineage 2. Genome accession: ERS23863191.

^*g*^ Marker for delamanid and pretomanid cross-resistance according to the second edition of the WHO mutation catalog (2).

^*h*^ AST, antimicrobial susceptibility testing; CFZ, clofazimine; DLM, delamanid; PMD, pretomanid; R, resistant; S, susceptible.

To complement these findings, we analyzed lineage 1 mutants that had been selected *in vitro* using clofazimine by Snobre et al. (9). During the first round of selection, an *Rv0678* frameshift was selected that conferred elevated bedaquiline and clofazimine MICs, whereas the delamanid and pretomanid MICs did not increase (Table 2) (2). Additional exposure to clofazimine selected *fbiC* Arg536Leu that correlated with cross-resistance to both delamanid and pretomanid using WHO critical concentrations (8, 10). Moreover, the acquisition of the *fbiC* mutation correlated with a clofazimine MIC increase by approximately a factor of two, which was in line with the magnitude reported by Waller et al. in an *Rv0678* wild-type background, whereas the bedaquiline MIC was not affected (Table 2).

**TABLE 2:** Antimicrobial susceptibility testing results of *in vitro* selected mutants following serial exposure to clofazimine by Snobre et al. (9)^*i*^

Isolate	Acquired mutations and allele frequency^*a*^	Broth microdilution MIC in µg/mL^*b*^				Categorical MGIT result^*c*^	
		BDQ	CFZ	DLM	PMD	DLM	PMD
2002-01038 (parental^*d*^)	None	0.06 (x1); 0.125 (x1)	0.125 (x3)	0.008 (x1); 0.016 (x1)	1 (x2)	S (x1)	Siu (x1)
2012-01331 (1st passage^*e*^)	*Rv0678* 344delA^*f*^ (0.9)	0.5 (x1); 1 (x1)	1 (x2); 2 (x1)	0.016 (x1)	0.5 (x1)	S (x1)	Siu (x1)
2013-02481 (2nd passage^*g*^)	*Rv0678* 344delA^*f*^ (1.0), *fbiC* Arg536Leu (1.0)	0.5 (x1); 1 (x1)	2 (x1); 4 (x2)	0.25 (x1)	>2 (x1)	R (x1)	R (x1)
H37Rv (control^*h*^)	None	0.125 (x2); 0.25 (x1)	0.06 (2x); 0.03 (x1)	0.008 (x3)	0.125 (x3)	S (x3)	S (x3)

^*a*^ The figures in parentheses correspond to the allele frequency.

^*b*^ The figures in parentheses correspond to the replicates from independent batches. No breakpoints currently exist to interpret broth microdilution MICs.

^*c*^ Only the current WHO critical concentrations (i.e., 0.06 µg/mL for delamanid and 0.5 and 2.0 µg/mL for pretomanid) were tested. Because the parental strain is lineage 1, it and the 1st passage mutant are intrinsically less susceptible to pretomanid (i.e., 0.5 < MIC ≤ 2 µg/mL in MGIT), which is interpreted as susceptible with interpretive uncertainty according to WHO, but susceptible according to EUCAST (8, 10).

^*d*^ Lineage 1. Genome accession: ERR13663834.

^*e*^ Selected by exposing 2002-01038 to 2 µg/mL clofazimine. Genome accession: ERR13663835.

^*f*^ Marker for bedaquiline and clofazimine cross-resistance according to the second edition of the WHO mutation catalog (2).

^*g*^ Selected by exposing 2012-01331 to 8 µg/mL clofazimine. Genome accession: ERR8025345. Previously published antimicrobial susceptibility testing results can be found in Table S1 (11–13).

^*h*^ The BCCM 500735 (equivalent to ATCC 27294) susceptible control strain was included in each round of testing.

^*i*^ BDQ, bedaquiline; CFZ, clofazimine; DLM, delamanid; PMD, pretomanid; R, resistant; S, susceptible; Siu, susceptible with interpretive uncertainty.

Our *in vitro* results demonstrate that clofazimine can select for cross-resistance to delamanid and pretomanid in three different lineages (i.e., 1, 2, and 4; Tables 1 and 2), but only after the acquisition of *Rv0678* mutations. This raises the possibility that the link between clofazimine and nitroimidazoles in a wild-type *Rv0678* background observed by Waller et al. might be due to their use of an auxotroph (4). In fact, the twofold MIC increase reported by Waller et al. contrasts with the hypersusceptibility observed by Gurumurthy et al. for a *fbiC* knockout mutant of *M. bovis* Bacillus Calmette-Guérin (4, 14, 15). Data from clinical strains are needed to investigate this contradiction. For example, clofazimine MIC testing of *Rv0678* wild-type clinical strains with nitroimidazole cross-resistance caused by *fbiA*, *fbiB*, *fbiC*, *fbiD*, or *fgd1* mutations could be carried out (e.g., using some of the mutants known to have arisen prior to the clinical use of either nitroimidazole [16, 17]). Moreover, relevant insights might be gained by studying serial cultures from patients treated with clofazimine as part of regimens without nitroimidazoles, such as historical regimens prior to the approval of nitroimidazoles or the recently WHO-recommended 9 month all-oral regimen of bedaquiline, linezolid, levofloxacin, clofazimine, and pyrazinamide (1).

In light of these uncertainties, we support the current WHO classification of *fbiA*, *fbiB*, *fbiC*, *fbiD*, and *fgd1* as tier 2 genes for clofazimine that require ongoing monitoring (i.e., not tier 1 resistance mechanisms [2]). In fact, even if data from clinical strains confirmed that the five nitroimidazole resistance genes confer an approximately twofold MIC increase to clofazimine, we believe that patients with rifampicin-resistant tuberculosis for whom other regimens are not an option should not be deprived of clofazimine. This should remain the case until strong clinical and/or pharmacokinetic and pharmacodynamic evidence becomes available that these mechanisms actually lead to worse treatment outcomes (e.g., because clofazimine takes several weeks to achieve steady state using the current dosing that was not optimized for treating tuberculosis [18, 19]). WHO used a similar rationale in its first edition of the mutation catalog (20, 21). Specifically, WHO decided not to classify the *eis* G−10A, C−12T, and G−37GT promoter mutations as amikacin resistance mutations as they only confer approximately twofold MIC increases, whereas it considered the fourfold MIC increase conferred by *eis* C−14T and *rrs* C1402T to be likely clinically significant for the current amikacin dose of 15–20 mg/kg/day (i.e., these mutations had not been considered amikacin resistance mechanisms prior to that [1, 2, 20, 22]). Similarly, Pandey et al. also did not consider *ndh* to be a resistance mechanism for delamanid, ethionamide, isoniazid, or pretomanid (23).

In summary, the findings from this and related studies underline the value of conducting selection experiments using varied conditions, as these may yield unexpected results. The selection of nitroimidazole resistance by clofazimine needs to be further investigated and monitored in the clinical setting. Based on the available data, we propose that, in the rare event that nitroimidazole resistance mutations in *fbiA*, *fbiB*, *fbiC*, *fbiD*, or *fgd1* are found in a *pepQ* and *Rv0678* wild-type background, this nitroimidazole resistance should not be an exclusion criterion for the use of clofazimine (16, 24). *Ddn*-mediated nitroimidazole resistance has been described to be frequent in at least one setting but does not impact the clofazimine MIC according to Waller et al. (4, 25). Therefore, the detection of *Rv0678* resistance mutations will likely be the most frequent genotypic reason for not using clofazimine in practice.

## SUPPLEMENTAL MATERIAL

Supplemental material aac.01113-25-s0001.pdf Supplementary methods; Table S1.

ASM does not own the copyrights to Supplemental Material that may be linked to, or accessed through, an article. The authors have granted ASM a non-exclusive, world-wide license to publish the Supplemental Material files. Please contact the corresponding author directly for reuse.
