# Supplementary material for: In vitro exposure to clofazimine can select for delamanid and pretomanid resistance in Mycobacterium tuberculosis
Source: Antimicrob Agents Chemother. 2025 Sep 22;69(11):e01113-25. doi: 10.1128/aac.01113-25 (PMC12587575; doi:10.1128/aac.01113-25)
Supplement: Supplemental material — Supplementary methods; Table S1. [file aac.01113-25-s0001.pdf]

Methods for testing in South Africa of mutants by Ismail et al.

MIC testing by MGIT

Clofazimine (C8895, Sigma-Aldrich Co., St Louis, USA), delamanid (NR-51636, BEI Resources, NIH/NIAID, USA) and pretomanid (RTI#11356-156, Mylan, West Virginia, USA) were formulated in DMSO (41639, Sigma-Aldrich Co.) to stock concentrations of 1 mg/ml and maintained at −20 °C (max: 6 months). Two-fold dilutions with a final concentration ranging from 4 to 0.06 µg/ml (clofazimine), 1 to 0.016 µg/ml (delamanid) and 2 to 0.016 µg/ml (pretomanid) were prepared from the stock solutions. An 8 µg/ml clofazimine concentration could not be included as the color of the drug solution interfered with the florescent detection of the BACTEC MGIT960 instrument. MIC determinations were performed as previously described (1). A 1:5 dilution of a three- to five-day positive liquid culture was used to inoculate (500 µl) MGIT tubes each containing the above-described clofazimine, delamanid and pretomanid concentrations. A further 1:100 dilution of the 1:5 suspension was used to inoculate (500 µl) a drug-free MGIT control tube. A H37Rv strain was included in each batch of MIC determinations conducted. Tubes were incubated until the growth control reached 400 growth units or for a maximum of 28 days. The MIC value was defined as the lowest drug concentration at which bacterial growth was inhibited and interpreted according to the latest WHO critical concentrations (2, 3).

Methods for testing in Belgium of mutants by Snobre et al.

MIC testing by broth microdilution

Stock solutions of bedaquiline, clofazimine, pretomanid, and delamanid were prepared in DMSO. Inner wells of the sterile, 96-well, round bottom polystyrene microtiter plates were filled manually with 100 µl of 7H9-S medium (7H9 broth + 10% OADC + 0.5% glycerol + 0.1% casitone), and outer wells with sterile distilled water (SDW) or 7H9-S.

Serial two-fold dilutions of clofazimine (0.016–4.0 µg/ml), bedaquiline (0.008–2.0 µg/ml), pretomanid (0.008–2.0 µg/ml), and delamanid (0.002–0.5 µg/ml) were distributed directly in the 7H9-S wells using the HP D300e dispenser. The final DMSO concentration was normalized to 0.2% across all wells containing DMSO-dissolved drugs, as well as DMSO-containing positive and negative controls, except for the well with 4 µg/ml clofazimine, which contained 0.4% DMSO.

A sterile loop was used to transfer bacterial colonies from 2–3 weeks old *M. tuberculosis* cultures grown on Löwenstein-Jensen (LJ) medium into a 15 ml sterile screw-cap glass tube containing 5–10 sterile glass beads. About three drops of SDW were added to this tube, which was then firmly closed and vortexed vigorously for one minute, paying attention to the beads rolling down the glass tube's wall, until the clumps were well dispersed. After allowing the closed tube to stand for 5 minutes to settle aerosols, 5 ml of SDW was added, the tube was tightly closed, and the contents were vigorously vortexed for 15 seconds until the tube's content was homogenized. After letting the closed tube stand for 30 minutes for the clumps to settle, the supernatant was transferred into a new sterile glass tube. Using the densitometer, the turbidity of the supernatant was adjusted to McFarland standard (McF) 0.5 using SDW, and then further diluted 1:100 in 7H9-S broth to prepare a 10-2 of the McF 0.5 bacterial suspension.

After inoculation, the plates were incubated at 36°C (±2°C) for a maximum of 21 days. The plates were read by visual inspection using an inverted mirror to detect growth in the wells. Systematic reading was done at day 7 and day 14 of incubation. If there was still no growth of the GC1% after day 14, the incubation was extended to a maximum of 21 days. As soon as the growth GC100% and the GC1% were positive, or when at least two of the three the GC1% positive controls of the same isolate were positive, the MICs were interpreted.

AST in MGIT

Delamanid (8,000 µg/ml) and pretomanid (4,000 µg/ml) stock solutions were prepared by dissolving the drug powders in DMSO. An inoculum was prepared directly from a positive MGIT tube that had flagged within 1–2 day or after a 1 in 5 dilution of a positive MGIT tube that had flagged within 3–5 days, and 500 µL of inoculum was added to the drug-containing tubes supplemented with 800 µL of OADC. The drug-free control vial was inoculated with a 1:100 dilution of the inoculum. MICs were determined using MGIT 960 standalone protocol according to the manufacturer's instructions. The critical concentrations used were 0.06 µg/ml for delamanid and 0.5 and 2.0 µg/ml for pretomanid according to the latest WHO critical concentration (3).

Bioinformatic methods

Mutations in clofazimine and nitroimidazole resistance genes according to the second edition of the WHO mutation catalogue shown in Tables 1 and 2 were called using version 6.6.3 of TB Profiler using its standard settings from Illumina whole genome sequencing data (4, 5). Mutation that failed quality control were not included.

Table S1

Previous published AST results for 2013-02481 from Table 2.

Drug	MIC (in µg/ml) followed interpretation in parentheses[a]					
	UKMYC5	EUCAST	REMA	7H10	7H11	MGIT
**Bedaquiline**	0.5 x22 (R),<br>≥1 x5 (R)	0.25[b]	0.25		0.5 (R)	R[b,c]<br>R[c]
**Clofazimine**	0.25 x4 (S),<br>0.5 x6 (R),<br>1 x12 (R),<br>2 x2 (R),<br>>4 x3 (R)	4[b]	1	0.5		R[b,c],<br>R[c]
**Delamanid**	0.12 x8 (S),<br>0.25 x9 (R),<br>0.5 x9 (R),<br>>1 (R)	0.5[b]	2		0.25 (R)	R[b,c],<br>S[c]
**Pretomanid**		>2[b]			>8[d]	R[b,c],<br>>4 (R)[d]

[a] Unless otherwise stated, testing was done once, and the results were from Rancoita et al., where 2013-02481 corresponds to the duplicate strains CRY-9 and CRY-22 (6). Where available WHO breakpoints were used for the interpretation (3). UKMYC5 were interpreted according to breakpoints that are not endorsed by WHO and have been questioned (7–9).

[b] 2013-02481 corresponds to strain XTB_16-005 from Mansjö et al. (10).

[c] Only the WHO breakpoint was tested (3).

[d] From Rupasinghe et al. (11).

References

1. Ismail N, Omar SV, Ismail NA, Peters RPH. 2018. *In vitro* approaches for generation of *Mycobacterium tuberculosis* mutants resistant to bedaquiline, clofazimine or linezolid and identification of associated genetic variants. J Microbiol Methods 153:1–9.

2. Sirgel FA, Wiid IJF, Helden PD van. 2008. Measuring minimum inhibitory concentrations in mycobacteria. Methods Mol Biol 465:173–186.

3. World Health Organization. 2024. WHO operational handbook on tuberculosis: module 3: diagnosis: rapid diagnostics for tuberculosis detection: web annex C: technical manual for culture-based drug susceptibility testing of anti-tuberculosis drugs used in the treatment of tuberculosis, 3rd ed. https://iris.who.int/handle/10665/376286. Retrieved 25 March 2024.

4. Phelan JE, O'Sullivan DM, Machado D, Ramos J, Oppong YEA, Campino S, O'Grady J, McNerney R, Hibberd ML, Viveiros M, Huggett JF, Clark TG. 2019. Integrating informatics tools and portable sequencing technology for rapid detection of resistance to anti-tuberculous drugs. Genome Med 11:41.

5. World Health Organization. 2023. Catalogue of mutations in *Mycobacterium tuberculosis* complex and their association with drug resistance, 2nd ed. https://iris.who.int/handle/10665/374061. Retrieved 24 February 2024.

6. Rancoita PMV, Cugnata F, Cruz ALG, Borroni E, Hoosdally SJ, Walker TM, Grazian C, Davies TJ, Peto TEA, Crook DW, Fowler PW, Cirillo DM, Crook DW, Peto TEA, Walker AS, Hoosdally SJ, Cruz ALG, Grazian C, Walker TM, Fowler PW, Wilson D, Clifton D, Iqbal Z, Hunt M, Smith EG, Rathod P, Jarrett L, Matias D, Cirillo DM, Borroni E, Battaglia S, Chiacchiaretta M, Filippo MD, Cabibbe A, Tahseen S, Mistry N, Nilgiriwala K, Chitalia V, Ganesan N, Papewar A, Rodrigues C, Kambli P, Surve U, Khot R, Niemann S, Kohl T, Merker M, Hoffmann H, Lehmann S, Plesnik S, Ismail N, Omar SV, Joseph L, Marubini E, Thwaites G, Thuong TNT, Ngoc NH, Srinivasan V, Moore D, Coronel J, Solano W, He G, Zhu B, Zhou Y, Ma A, Yu P, Schito M, Claxton P, Laurenson I. 2018. Validating a 14-drug microtiter plate containing bedaquiline and delamanid for large-scale research susceptibility testing of *Mycobacterium tuberculosis*. Antimicrob Agents Chemother 62:10.1128/aac.00344-18.

7. CRyPTIC. 2022. Epidemiological cutoff values for a 96-well broth microdilution plate for high-throughput research antibiotic susceptibility testing of *M. tuberculosis*. European Respir J 2200239.

8. Kahlmeter G, Turnidge J. 2023. The determination of epidemiological cut-off values requires a systematic and joint approach based on quality controlled, non-truncated minimum inhibitory concentration series. Eur Respir J 61:2202259.

9. Köser CU, Maurer FP. 2023. Minimum inhibitory concentrations and sequencing data have to be analysed in more detail to set provisional epidemiological cut-off values for *Mycobacterium tuberculosis* complex. Eur Respir J 61:2202397.

10. Mansjö M, Espinosa-Gongora C, Samanci I, Groenheit R, Werngren J. 2025. Performance of a broth microdilution assay for routine minimum inhibitory concentration determination of 14 anti-tuberculous

drugs against the *Mycobacterium tuberculosis* complex based on the EUCAST reference protocol. Antimicrob Agents Chemother 69:e00946-24.

11. Rupasinghe P, Reenaers R, Vereecken J, Mulders W, Cogneau S, Merker M, Niemann S, Omar SV, Rigouts L, Köser CU, Decroo T, Jong BC de. 2024. Refined understanding of the impact of the *Mycobacterium tuberculosis* complex diversity on the intrinsic susceptibility to pretomanid. Microbiol Spectr e0007024.
